# Concurrent Thyroiditis and Hypophysitis in a Patient Receiving Dual Immune Checkpoint Inhibitor Therapy for Metastatic Melanoma

**DOI:** 10.7759/cureus.89689

**Published:** 2025-08-09

**Authors:** Hima Darapu, Aryn E Kormanis

**Affiliations:** 1 Endocrinology, Diabetes and Metabolism, Wake Forest School of Medicine, Winston-Salem, USA

**Keywords:** central adrenal insufficiency, endocrinopathies, hypophysitis, hypopituitarism, immune checkpoint inhibitors, immune checkpoint inhibitors induced endocrinopathies, thyroiditis

## Abstract

Immune checkpoint inhibitors (ICIs), particularly when used in combination regimens, are associated with a broad range of immune-related adverse events (irAEs), including endocrinopathies. We present a case of a 53-year-old male patient with metastatic melanoma treated with a combination of ipilimumab and nivolumab who developed concurrent thyroiditis and hypophysitis. Although this co-occurrence is uncommon, it is clinically significant. A key learning point from this case is the importance of accurate interpretation of laboratory findings. Our patient was transitioning from the hyperthyroid phase of subacute thyroiditis to hypothyroidism and simultaneously developed central hypothyroidism due to hypophysitis. This overlap created a diagnostic challenge that could have been misinterpreted without careful correlation of the laboratory findings, a solid understanding of how ICIs can cause diverse endocrinopathies, and awareness of the typical phases of thyroiditis. Clinical context played a critical role in correctly identifying concurrent thyroiditis and central hypothyroidism due to hypophysitis. This case highlights the importance of timely recognition of ICI-induced endocrinopathies and careful interpretation of labs, especially when multiple endocrinopathies coexist.

## Introduction

Immune checkpoint inhibitors (ICIs) such as ipilimumab (a cytotoxic T-lymphocyte-associated protein 4 or CTLA-4 inhibitor) and nivolumab (a programmed cell death protein 1 or PD-1 inhibitor) are effective therapies for advanced melanoma but are associated with immune-related adverse events (irAEs), including endocrinopathies, many of which are irreversible [1,2]. Severe or life-threatening events occur in up to 55% of patients receiving dual-agent regimens [3]. Among endocrine irAEs, thyroiditis is most commonly linked to PD-1 inhibitors [1,2,4], while hypophysitis is more frequently associated with CTLA-4 blockade [1,2,5]. Though each of these toxicities is well-documented, their concurrent presentation is rare and not well characterized [6]. 

We report a case of a patient treated with dual ICIs who developed both thyroiditis and hypophysitis within a narrow time window, presenting a diagnostic challenge that could have led to misinterpretation without careful clinical correlation and laboratory analysis. While multiple guidelines exist for the management of immunotherapy toxicities, navigating these recommendations and recognizing complex immunotoxicity patterns can be difficult for clinicians who are not familiar with their nuances [3]. This case highlights the importance of understanding the typical time course of ICI-induced endocrinopathies and interpreting endocrine lab values in a clinical context, particularly when overlapping dysfunctions exist. 

Evolving thyroiditis and central hypothyroidism from hypophysitis can occur simultaneously during ICI therapy, creating misleading laboratory findings especially if central hypothyroidism arises during the hypothyroid phase of thyroiditis [6]. This overlap can obscure the diagnosis of life-threatening but treatable conditions such as secondary adrenal insufficiency associated with hypophysitis. This case serves as a learning opportunity to prevent misdiagnosis of the concurrent development of ICI endocrinopathies as they can be life-threatening, but are treatable with replacement of the lacking endogenous hormone [3]. By illustrating these complexities, this case highlights the need for clinical vigilance, multidisciplinary coordination, and prompt hormone replacement when endocrine irAEs are suspected. 

## Case presentation

A 53-year-old male patient with newly diagnosed metastatic melanoma (BRAF V600E-positive), with the disease involving the lungs, bones, lymph nodes, and possibly the small bowel, was started on combination ICI therapy with ipilimumab and nivolumab. Baseline thyroid function was within normal limits before starting the immunotherapy (Table 1). 

**Table 1:** Laboratory trends demonstrating the transition from thyroiditis to panhypopituitarism during the immune checkpoint inhibitor (ICI) therapy Laboratory values collected at sequential time points during ICI therapy. Initial results show suppressed TSH and elevated free thyroxine (T4), consistent with the thyrotoxic phase of ICI-induced thyroiditis. This was followed by normalization of free T4 with continued TSH suppression, suggesting the transition to the euthyroid phase. Subsequent testing three weeks after cycle 3 revealed undetectable free T4 with inappropriately normal TSH, low cortisol, low testosterone, and inappropriately normal gonadotropins, consistent with evolving panhypopituitarism due to hypophysitis. Missing values (–) were not obtained at earlier time points. Baseline cortisol, adrenocorticotropic hormone (ACTH), and gonadal hormone levels (follicle stimulating hormone (FSH), luteinizing hormone (LH), testosterone) were not assessed at initial presentation.

Component (Reference range)	Baseline (Cycle 1)	At thyroiditis (Cycle 2)	Euthyroid (Cycle 3)	At hypophysitis (after Cycle 3)
Thyroid stimulating hormone or TSH (0.45-5.33 uIU/mL)	1.864	<0.05	<0.05	2.068
Free T4 (0.6-1.3 ng/dL)	0.8	3.1	0.9	<0.4
Adrenocorticotropic hormone (7.2-63.0 pg/mL)	-	-	-	5.8
Cortisol (6.7-22.6 mcg/dL)	-	-	-	2.1
Luteinizing hormone (1.2-8.6 mIU/mL)	-	-	-	2
Follicle-stimulating hormone (1.3-19.3 mIU/mL)	-	-	-	4.4
Testosterone (150-684 ng/dL)	-	-	-	36
Prolactin (2.6-13.1 ng/mL)	-	-	-	4.4

**Figure 1:**
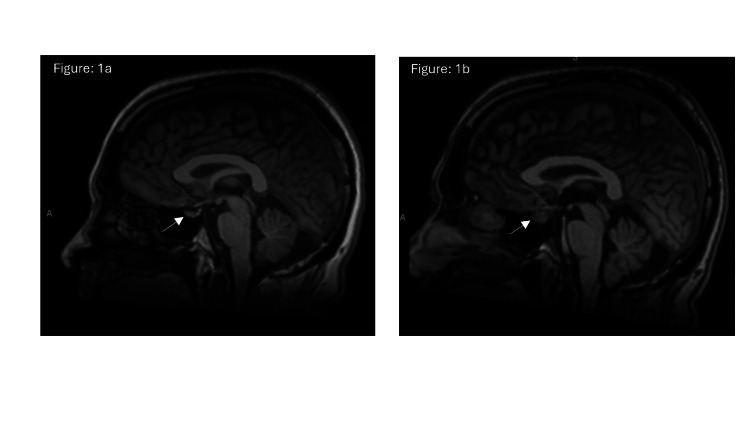
Sagittal T1-weighted post-contrast MRI images of the brain (1a) Represents baseline imaging prior to immune checkpoint inhibitor (ICI) therapy showing a normal-sized pituitary gland. (1b) Shows interval enlargement and heterogeneous contrast enhancement of the pituitary gland consistent with immune-related hypophysitis. Findings supported pituitary inflammation associated with combination ICI therapy.

**Figure 2:**
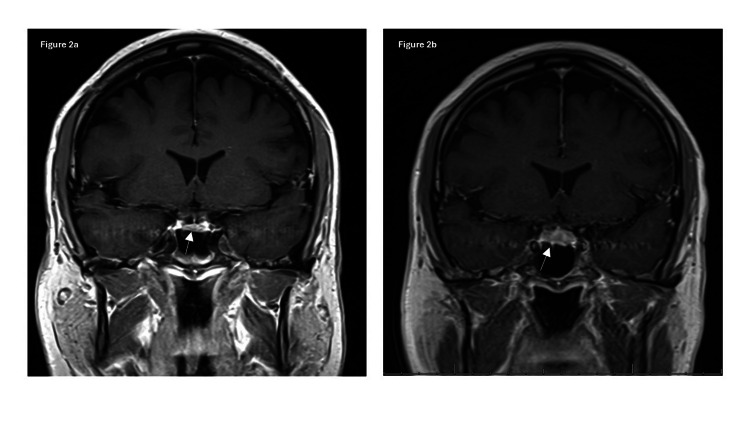
Coronal T1-weighted post-contrast MRI brain images (2a) Showing normal pituitary morphology at baseline (2b) Interval enlargement and enhancement after starting immune checkpoint inhibitor (ICI) therapy during symptomatic presentation of hypophysitis. This view complemented the sagittal imaging and demonstrated thickening of the pituitary stalk and gland expansion.

Before the second cycle of ICI, the thyroid labs showed a suppressed thyroid-stimulating hormone (TSH) and elevated free thyroxine (free T4), consistent with a thyrotoxic phase of ICI-induced thyroiditis. By the third cycle of ICI, TSH remained suppressed while free T4 had normalized, suggesting a transition to the hypothyroid phase (Table 1). Ten days after the third cycle, the patient began experiencing persistent, worsening headaches that were unresponsive to acetaminophen or opioids. Associated symptoms included low-grade fevers, fatigue, and photosensitivity. A non-contrast head CT was unremarkable except for mild mucosal thickening in the paranasal sinuses. He was treated with a short steroid course and empiric antibiotics for suspected sinusitis, with partial symptom relief. The headache recurred after steroid tapering, prompting an empiric one-time dose of solumedrol 125 mg IV, emergency evaluation, and hospital admission. Brain MRI demonstrated interval enlargement and contrast enhancement of the pituitary gland, consistent with hypophysitis (Figures 1b, 2b).

**Figure 3:**
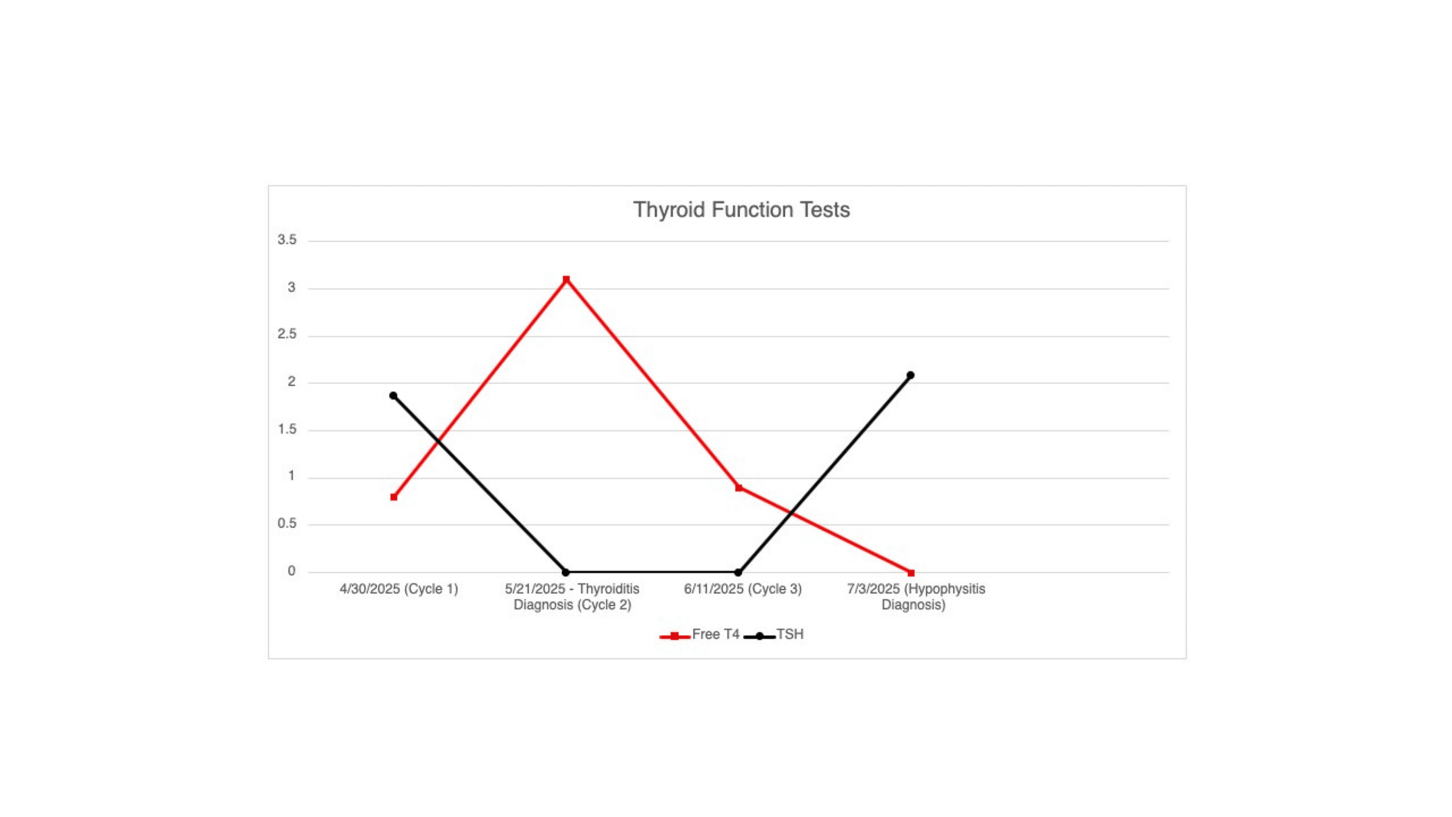
Serial trends in thyroid-stimulating hormone (TSH) and free thyroxine (T4) during immune checkpoint inhibitor (ICI) therapy Line graph depicting serial thyroid function trends in the patient receiving combination ipilimumab and nivolumab. The graph illustrates the initial thyrotoxic phase (suppressed TSH, elevated Free T4), followed by a transient euthyroid period, and a subsequent decline in free T4 with inappropriately normal TSH indicative of central hypothyroidism. These changes correlated with the onset of hypophysitis diagnosed on MRI. Time is plotted in weeks from the initiation of ICI therapy. All values were obtained prior to the initiation of thyroid hormone replacement. TSH is measured in uIU/mL and free T4 in ng/dL.

Laboratory testing revealed an inappropriately normal TSH with an undetectable free T4, raising concern for central hypothyroidism (Figure 3).

Morning cortisol was low, although the interpretation was limited by prior corticosteroid exposure. Gonadotropins were inappropriately normal in the context of low testosterone, further supporting the diagnosis of panhypopituitarism (Table 1). 

The patient was initiated on sick day dosing hydrocortisone replacement (30 mg in the morning and 10 mg in the evening; transitioned to a physiological maintenance dose of 10 mg in the morning and 5 mg in the evening upon discharge) and levothyroxine for central hypothyroidism with significant symptom improvement. Due to the development of endocrinopathies, combination ICI therapy was suspended. Imaging following three cycles of ipilimumab and nivolumab demonstrated a partial response of the melanoma, and maintenance nivolumab monotherapy was planned for re-initiation at the outpatient oncology follow-up. The patient continued endocrine follow-up for titration of hormone replacement therapy. 

## Discussion

ICIs have revolutionized the treatment of advanced malignancies by enhancing T-cell-mediated immune responses against tumors, including Ipiplimumab, a CTLA-4 inhibitor, first approved for treatment of metastatic melanoma in 2011 [7,8]. However, their use is associated with a range of irAEs, among which endocrinopathies are both common and clinically significant [3]. The incidence of endocrine irAEs varies depending on the specific ICI used. Thyroid dysfunction occurs in up to 20% of patients receiving PD-1 or programmed death-ligand 1 (PD-L1) inhibitors [2], while hypophysitis is more commonly seen with CTLA-4 inhibitors, affecting approximately 10% of treated individuals [1]. 

The risk of irAEs, including endocrinopathies, increases substantially with combination ICI therapy [9,10]. Studies have shown that dual checkpoint blockade (e.g., ipilimumab plus nivolumab) results in a higher incidence of endocrine toxicities compared to monotherapy [8]. Hypophysitis specifically is associated with an incidence rate of 6.4% while on combination therapy versus <0.1% to 3.2% on various monotherapy regimens [8,11]. The average time from ICI initiation to development of an endocrinopathy has been found to be 4.1 +/- 2.8 months [2], emphasizing the importance of hormone level screening with each cycle of therapy. ICI therapy has also been associated with the incidence of multisystem irAEs of approximately 15%, the most significant was the overlap of myositis, myocarditis, and myasthenia gravis [7]. There has been no data published on the simultaneous occurrence of two distinct endocrinopathies [7]. 

Reports of concurrent immune-related thyroiditis and hypophysitis are rare, with very few cases cited as of May 2025 [6], but it is increasingly recognized as checkpoint inhibitor use expands. The coexistence of these two distinct endocrinopathies likely reflects a cumulative immune-mediated effect triggered by the simultaneous blockade of both CTLA-4 and PD-1 pathways. There is a single report of a triple endocrinopathy effect of ICI therapy, which included thyroiditis, hypophysitis, and adrenalitis [12]. Although rare, these cases highlight the importance of continued laboratory monitoring and maintaining a high index of suspicion even after the initial diagnosis of endocrinopathy. Overlapping or sequential endocrine dysfunction can obscure clinical interpretation, increasing the already challenging course of irAEs, given their unpredictable onset and organ involvement [7]. For instance, central hypothyroidism may be mistaken for the resolution of thyroiditis. Without considering the possibility of central hypothyroidism, clinicians may overlook secondary adrenal insufficiency and other pituitary hormone deficiencies, ultimately delaying the recognition and diagnosis of hypophysitis. 

Furthermore, features of hypophysitis such as adrenal insufficiency and hypogonadotropic hypogonadism often manifest with vague symptoms including fatigue, headache, and electrolyte abnormalities. These can be mistakenly attributed to the malignancy itself or treatment-related side effects if not thoroughly investigated. This is evidenced by documented presentations for symptoms such as fever, nausea, fatigue, and lethargy [8,12]. 

In addition to its often subtle and nonspecific clinical presentation, immune-related hypophysitis can be radiologically elusive. The MRI frequently appears normal or shows only nonspecific pituitary changes. In our case, pituitary enlargement and enhancement were evident on MRI, helping to support the diagnosis. However, such findings are not consistently present. A 2023 study evaluating hypophysitis and hypopituitarism in patients receiving ICIs found that only two of the nine patients had radiographic evidence of pituitary enlargement [13]. Pituitary enlargement, raising radiologic findings for hypophysitis, are more commonly seen in patients on combination ICI therapy and is often accompanied by mass effect symptoms, commonly characterized as a headache or diplopia [11]. The infrequent appearance of definitive MRI findings complicates efforts to establish the timeline of pituitary involvement and symptom onset, reinforcing the importance of clinical suspicion and biochemical evaluation in diagnosis. 

A key learning point from this case is the importance of accurately interpreting endocrine laboratory values, particularly during the dynamic phases of immune-related thyroiditis. Our patient was transitioning from the thyrotoxic phase to the hypothyroid phase of PD-1 inhibitor-induced thyroiditis when he concurrently developed central hypothyroidism due to ICI-induced hypophysitis. Typically, in primary hypothyroidism, a low free T4 would be accompanied by an elevated TSH. However, in this case, the TSH was inappropriately normal despite an undetectable free T4, raising concern for central hypothyroidism. This finding, when interpreted alongside brain MRI revealing pituitary enlargement and enhancement, supported the diagnosis of hypophysitis. Recognizing this pattern is critical, particularly during the transition from thyroiditis-induced thyrotoxicosis to hypothyroidism. It is important to maintain a high index of suspicion and routinely evaluate both TSH and free T4 in patients on ICI therapy when endocrine dysfunction is suspected, to ensure timely diagnosis and initiation of hormone replacement. 

This case highlights the need for a high index of suspicion for concurrent endocrinopathies in patients receiving combination ICI therapy. Multidisciplinary coordination, prompt hormonal evaluation, and careful interpretation of laboratory findings in their clinical context are crucial to minimizing morbidity associated with immune-related endocrine toxicities. 

## Conclusions

This case highlights the rare co-occurrence of thyroiditis and hypophysitis in a patient receiving combination ICI therapy. An inappropriately normal TSH despite a low free T4 during the expected transition from thyrotoxicosis to hypothyroidism served as a critical clue to central hypothyroidism. Brain MRI showed pituitary enlargement and enhancement, further supporting the diagnosis of hypophysitis. Early recognition allowed prompt initiation of hydrocortisone and levothyroxine therapy, leading to significant symptom improvement. Recognizing these laboratory patterns is crucial to avoid delays in identifying life-threatening but treatable conditions such as secondary adrenal insufficiency. This case emphasizes the importance of timely lab interpretation, appropriate imaging, and early hormone replacement in optimizing outcomes for patients with ICI-induced endocrinopathies.

## References

[1] Wright JJ, Powers AC, Johnson DB (2021). Endocrine toxicities of immune checkpoint inhibitors. Nat Rev Endocrinol.

[2] Elshafie O, Khalil AB, Salman B, Atabani A, Al-Sayegh H (2024). Immune checkpoint inhibitors-induced endocrinopathies: assessment, management and monitoring in a comprehensive cancer centre. Endocrinol Diabetes Metab.

[3] Darnell EP, Mooradian MJ, Baruch EN, Yilmaz M, Reynolds KL (2020). Immune-related adverse events (irAEs): diagnosis, management, and clinical pearls. Curr Oncol Rep.

[4] Iwama S, Kobayashi T, Yasuda Y, Arima H (2022). Immune checkpoint inhibitor-related thyroid dysfunction. Best Pract Res Clin Endocrinol Metab.

[5] Michot JM, Bigenwald C, Champiat S (2016). Immune-related adverse events with immune checkpoint blockade: a comprehensive review. Eur J Cancer.

[6] Zavgorodneva Z, Khan M, Guber H (2025). Co-occurrence of hypophysitis and thyroiditis induced by PD-L1 inhibitor avelumab: clinical insights. JCEM Case Rep.

[7] Gougis P, Jochum F, Abbar B (2024). Clinical spectrum and evolution of immune-checkpoint inhibitors toxicities over a decade-a worldwide perspective. EClinicalMedicine.

[8] Barnabei A, Carpano S, Chiefari A (2020). Case report: ipilimumab-induced panhypophysitis: an infrequent occurrence and literature review. Front Oncol.

[9] Li Z, Liu Z, Wei H, Jin S, Sun Y, Zhang Y, Liu Y (2025). Risk of pituitary immune-related adverse events caused by immune checkpoint inhibitors: a systematic review and meta-analysis. Endocr Pract.

[10] Aldrete K, Shahla L (2024). Simultaneous hypophysitis with adrenal crisis and thyroiditis due to immunotherapy: a case report. Endocr Pract.

[11] Johnson J, Goldner W, Abdallah D, Qiu F, Ganti AK, Kotwal A (2023). Hypophysitis and secondary adrenal insufficiency from immune checkpoint inhibitors: diagnostic challenges and link with survival. J Natl Compr Canc Netw.

[12] Rossi S, Silvetti F, Bordoni M, Ciarloni A, Salvio G, Balercia G (2024). Pembrolizumab-induced thyroiditis, hypophysitis and adrenalitis: a case of triple endocrine dysfunction. JCEM Case Rep.

[13] Chiloiro S, Giampietro A, Bianchi A (2023). Pituitary enlargement and hypopituitarism in patients treated with immune checkpoint inhibitors: two sides of the same coin?. J Pers Med.

